# Service providers’ use of harm reduction approaches in working with older adults experiencing abuse: a qualitative study

**DOI:** 10.1186/s12877-021-02328-1

**Published:** 2021-06-30

**Authors:** Donna Goodridge, Kerstin Stieber Roger, Christine A. Walsh, Elliot PausJenssen, Marina Cewick, Carla Liepert

**Affiliations:** 1 College of Medicine, University of Saskatchewan, Saskatoon, Canada; 2 Department of Community Health Sciences, Rady Faculty of Health Sciences, University of Manitoba, Winnipeg, Canada; 3 Faculty of Social Work, University of Calgary, Calgary, Canada; 4 Saskatoon Council on Aging, Saskatoon, Saskatchewan Canada; 5 Faculty of Education, University of Manitoba, Winnipeg, Canada

**Keywords:** Elder abuse, Elder mistreatment, Harm reduction, Service providers, Thematic analysis

## Abstract

**Background:**

Although abuse experienced by older adults is common and expected to increase, disclosure, reporting and interventions to prevent or mitigate abuse remain sub-optimal. Incorporating principles of harm reduction into service provision has been advocated as a strategy that may improve outcomes for this population. This paper explores whether and how these principles of harm reduction were employed by professionals who provide services to older adults experiencing abuse.

**Methods:**

Thematic analysis of qualitative interviews with 23 professionals providing services to older adults experiencing abuse across three Western provinces of Canada was conducted. Key principles of harm reduction (humanism, incrementalism, individualism, pragmatism, autonomy, and accountability without termination) were used as a framework for organizing the themes.

**Results:**

Our analysis illustrated a clear congruence between each of the six harm reduction principles and the approaches reflected in the narratives of professionals who provided services to this population, although these were not explicitly articulated as harm reduction by participants. Each of the harm reduction principles was evident in service providers’ description of their professional practice with abused older adults, although some principles were emphasized differentially at different phases of the disclosure and intervention process. Enactment of a humanistic approach formed the basis of the therapeutic client-provider relationships with abused older adults, with incremental, individual, and pragmatic principles also apparent in the discourse of participants. While respect for the older adult’s autonomy figured prominently in the data, concerns about the welfare of the older adults with questionable capacity were expressed when they did not engage with services or chose to return to a high-risk environment. Accountability without termination of the client-provider relationship was reflected in continuation of support regardless of the decisions made by the older adult experiencing abuse.

**Conclusions:**

Harm reduction approaches are evident in service providers’ accounts of working with older adults experiencing abuse. While further refinement of the operational definitions of harm reduction principles specific to their application with older adults is still required, this harm reduction framework aligns well with both the ethical imperatives and the practical realities of supporting older adults experiencing abuse.

**Supplementary Information:**

The online version contains supplementary material available at 10.1186/s12877-021-02328-1.

## Background

Increasingly recognized as a pressing public health issue [1], abuse of older adults refers to action or lack of appropriate action within a relationship that causes harm or distress to older persons, including emotional/psychological abuse, financial/material abuse, sexual abuse and neglect [2]. A systematic review by Dong [3] found that elder abuse was associated with outcomes such as: psychological disturbance; poor health; increased emergency room visits and hospitalizations; premature nursing home placement; and shortened survival.

Risk factors for abuse of community-dwelling older adults identified in one systematic review [4] include characteristics of the: a) older adult (cognitive impairment; psychiatric illness; functional dependency; poor health or frailty; ethnicity; loneliness; alcohol use); b) perpetrator (caregiver burden, psychiatric illness; substance misuse; personality traits; ethnicity, cognitive impairment, trauma or past abuse); c) relationship between the older adult and perpetrator (family disharmony; poor or conflictual relationships); and d) environment (low levels of social support). Importantly, the risk factors with the highest odds ratios predicting abuse of older adults in this review were relationships and environment.

While the number of cases of abuse of community-residing older adults is expected to double in the coming 15–20 years based on the growing population of seniors [5], societal issues such as increased rates of poverty [6] and social isolation due to COVID-19 [7] may further increase the numbers of older adults at risk for abuse. In addition, the ripple effects of the opioid crisis on abuse of older adults, including theft of medications, financial exploitation, and physical abuse, also appear to be rapidly accelerating [8].

Detection and reporting of abuse of older adults, as well as interventions to prevent or mitigate abuse, however, remain sub-optimal [5, 9], in spite of strategies such as mandatory reporting in some jurisdictions [5]. Although estimates of the prevalence of abuse of community-dwelling older adults range from 1.1% [10] up to 25.3% [11], actual rates are suspected to be much higher [12]. For example, Lachs [6] found the older adult abuse rate in New York City to be 24 times greater than the number of cases referred to law enforcement, legal authorities or social services. The National Elder Mistreatment Study [13] reported that only 15.4% of older adults experiencing abuse sought help through the police or other authorities. A study of 2880 older women in five European countries found that 30.1% of older reported at least one experience of abuse in the previous year, and less than half talked about this in an informal setting or reported it to a formal agency [14].

Under-reporting results from both client- and provider-related factors. Abusive behaviors may not be recognized or defined as such by older adults [15, 16]; the older adult may be reluctant to place a relationship on which they are dependent at risk [17, 18]; there may be fear of institutionalization [4] and access to appropriate services may be limited [19–21]. Older adult victims of physical abuse, poly-victimization (multiple instances and types of abuse), or those in which a perpetrator had previous police involvement were found to be more likely to seek help, while help-seeking was lower among older adults who were dependent on their perpetrator or when the perpetrator had a large friendship network [13]. Providers may fail to recognize or know how to respond to suspected abuse of older adults, or feel reporting would adversely affect trust in the clinician-patient relationship [12, 22–24].

In order to address the challenges related to disclosure, reporting, and intervention, Burnes and colleagues [5] proposed a conceptual practice model of community elder mistreatment with capable older adults. The model’s principles of voluntariness, self-determination, and adopting the least restrictive path are intended to provide much-needed guidance for researchers and service providers. Harm reduction is advocated in this model as an orientation that can promote abused older adults’ self-determined and personal construction of a successful outcome, without seeking to completely eliminate or resolve the situation [5]. A harm reduction approach provides the opportunity for a range of solutions along the risk reduction continuum, while reconciling the fact that some older adults will choose to live in ways that may jeopardize their own safety [5].

Harm reduction is a public health strategy that aims to reduce the harms of usually stigmatized behaviors, recognizing that abolition may, in fact, exacerbate harms [25]. While typically associated with strategies to mitigate illicit drug use, the principles of harm reduction are being increasingly applied to diverse populations, including sex workers [26, 27], persons with eating disorders [28] and youth sex exploitation [29]. In fact, Hawk et al. [30] suggest that harm reduction should be considered a universal precaution that can be applied “to all individuals regardless of their disclosure of negative health behaviors, given that health behaviors operate along a continuum and are not binary” (pp. 7–8). Older adults experiencing abuse are often vulnerable individuals who make decisions that may jeopardize their own health and safety, suggesting that harm reduction may be a helpful lens through which to address these situations. Burnes et al. [5] advocated for the use of a harm reduction approach in cases of abuse of older adults, where mistreatment cases are typically never completely resolved, and where the severing of close, often familial relationships, would be discordant with the voluntary nature of requesting supportive services.

Hawk and colleagues [30] defined six comprehensive principles of harm reduction (humanism, pragmatism, individualism, autonomy, incrementalism and accountability without termination) that might be applied to working with older adults experiencing abuse. *Humanism* recognizes that harmful behaviors provide some advantage to the individual, which must be assessed and acknowledged to understand the balance between harms and benefits. Recognizing that positive change can take an extended time, and that plateaus and negative trajectories are common, the principle of *incrementalism* emphasizes the need for ongoing support in cases of abuse of older adults. Because older adults present on a spectrum of harm and receptivity to change, the principle of *individualism* in harm reduction requires the consideration of individual strengths and needs, as well as tailored messages and interventions. *Pragmatism* posits that abstinence (or severing the relationship, in the case of abused older adults) is neither prioritized nor assumed to be goal of the client. *Autonomy*, central to both the harm reduction approach and recognition of the older adult’s right to self-determination, is the harm reduction principle highlighting that individuals ultimately make their own choices. Provider-client relationships are characterized by shared decision-making, patient-driven care and reciprocal learning. Autonomy is evident in the finding that victims of elder abuse are generally unreceptive to the involvement of the criminal justice system [31]. Finally, *accountability without termination* highlights that, while older adults are responsible for their choices and behaviors and have the right to make harmful decisions, they should still be supported through the consequences of their decisions. Perceived “backwards movement” is not penalized by providers, who instead help patients to understand the impact of their choices and behaviors. This paper explores whether and how these principles of harm reduction were employed by professionals who provide service to older adults experiencing abuse.

## Methods

A larger study was conducted between March, 2019 and February, 2020 across three Prairie provinces in Canada (Manitoba, Saskatchewan and Alberta) with both older adults who experienced abuse as well as service providers. The objective of the overall study was to explore help-seeking, disclosure and reporting of abuse, and services available to older adults experiencing abuse. Ethical approval was granted by the University of Manitoba (#351893), University of Saskatchewan (#1957), and the University of Calgary (REB 19–0417). Because we collected data in three provinces, and studies of abuse of older adults were deemed “above minimal risk”, each local institution required separate ethical approval.

Participants were recruited from across each of the provinces through organizations and agencies that offered referrals or direct services to older adults experiencing abuse. These settings included urban, mixed urban-rural and rural agencies which provided: services to seniors that included abuse-related programs; abuse-specific intervention; general services for older adults unrelated to abuse; community services unrelated to specific services for older adults; home care services; hospitals; and police services. Recruitment posters in three time periods in 2019 were sent to social service and health care organizations and followed up by a telephone call to the manager/director of the organization to obtain a convenience sample.

The present paper focuses specifically on data elicited from service providers. Following written informed consent, data were collected by three research assistants (one in each province: MC, CL, KG) trained by the researchers (KR, CW, DG) in 23 individual face-to-face, telephone or virtual semi-structured, audiotaped interviews of 30–60 min with service providers. In order to obtain diverse perspectives, participants represented a broad range of professionals whose practice involved older adults experiencing abuse. Field notes were made during the interviews by the research assistants. Five broad interview questions were supplemented with probes and focused on capturing the experiences of providers in working with older adults experiencing abuse of any kind. In particular, the interview questions asked about: help-seeking and reporting abuse; the nature and quality of local services available to address abuse of older adults; and recommendations for assisting older adults who are considering reporting abuse. Direct questions about the use of harm reduction approaches were not included. Verbatim transcripts were prepared and identifying information was removed from the transcripts. Transcripts were returned for verification to participants.

Preliminary analysis of data from both older adults and providers from the larger study was conducted by the research team over three meetings. In order to more fully examine the data elicited from providers, these transcripts were uploaded into NVivo V. 12 and independently line-by-line coded by a researcher (DG) and a research associate (MR) using an inductive thematic analysis approach [32]. Themes were identified from the codes and reviewed, with emerging themes compared in relation to the codes and entire data set. The initial thematic analysis suggested that harm reduction approaches were embedded within the data, although were never explicitly articulated by participants. A literature search identified Hawk et al.’s [30] explication of harm reduction principles, which were used as a thematic framework for organizing the data.

Rigor [33] was supported throughout this study in several ways. Participants had the opportunity to review their transcripts and provide additional comments as desired. An audit trail ensured dependability, while independent coding, analysis and discussion allowed for confirmability. Transferability was achieved though rich textual description. This study was conducted in accordance with the Consolidated Criteria for Reporting Qualitative Research (Supplemental Material).

## Results

All participants (*N* = 23) were professionals currently working with older adults experiencing abuse. Fifteen social workers, four nurses, two program coordinators and two physicians took part in the interviews. Participants represented: agencies providing general services to seniors that included abuse-related programs (*n* = 8); agencies dedicated to abuse interventions (*n* = 3); community agencies offering intergenerational programs (*n* = 4); hospitals (*n =* 4); home care (*n* = 2) and police-based vulnerable persons services (*n =* 2). Fifteen participants were based in urban locations, two in rural areas and five provided services to both urban and rural areas.

The results of our analysis are presented below, with Hawk et al.’s [30] Harm Reduction principles used as primary themes to organize the sub-themes identified in the data.

### Humanism

Humanism describes the way providers respect, value, care for and dignify clients as individuals [28]. This approach was clearly reflected in multiple references about the importance of “seeing” the client holistically and approaching both disclosure and interventions related to abuse with compassion and understanding. Three sub-themes characterized the enactment of humanism by providers dealing with abused older adults: a) building a trusting relationship; b) understanding the context; and c) validating the older adult’s dignity and worth.

Participants were unanimous in their agreement that building a trusting relationship with the older adult experiencing abuse was foundational to both disclosure and intervention. Creating a safe space in which the older adult felt seen, heard and respected was typically the first step in building trust. “*Please know that we can have a safe conversation about that [behavior] and see what we can do to help you, at a pace that you are comfortable with, to a point that you’re comfortable with… and we do nothing unless you say it’s ok for us to help you with that*”. (Urban Community Seniors Program Coordinator) The skills of acceptance and being present to whatever the older adult wished to discuss fostered trust. “*It’s okay to air out your dirty laundry…it doesn’t mean you’re bad. Just means some really bad stuff is happening to you*.” (Urban Community Health Nurse).

Developing a relationship in which disclosure could take place often occurred over repeated encounters and was described as most effective when there was continuity in providers. “*There is kind of a worry of being kind of ‘tossed around’ from kind of support to support and not just having one main contact… a lot of what we hear is that we just want the one person for me to call.*” (Urban/rural Seniors’ Agency Coordinator) Several participants noted how difficult it was for older adults to have to repeat their story if a new provider became involved. “*Once they do that once, they maybe don’t want to do it again*” (Urban Seniors’ Agency Coordinator).

Humanism was also evident in efforts to situate the abuse within an understanding of the context of the older adult’s individual life circumstances. Generational differences in help-seeking were reported by some participants. “*We’ve got our older adult population and they’re not used to getting help. They’re not used to reaching out, they’re not the ones that go for counselling, and everything is very much kept in the family*.” (Urban/rural Seniors’ Agency Coordinator).

Supporting older adults experiencing abuse entailed validating their worth and rights as human beings, which had often been severely undermined in the context of abuse. “*You are worthy of good care. You are worthy of a good life. You deserve to have access to your finances in a healthy way to support you, your medication, your food, your life, your recreation. You deserve to not be abused*.” (Urban Community Health Nurse) Acknowledging the validity of the older adult’s experience was important in establishing a trusting relationship. “*For them to know that what they are feeling and what they are experiencing is real and it’s justified…whether it ends up that it’s abuse or something else, if it’s causing you distress, then you should feel that you can talk to someone - and it’s valid*.” (Urban/rural Seniors’ Agency Coordinator).

### Incrementalism

The harm reduction principle of incrementalism recognizes that positive steps may take months or years to achieve [30]. Small successes can promote ongoing engagement with service providers and provide for continued access in the event of future crises. Descriptions of incremental approaches adopted by participants were evident during both the disclosure and intervention processes.

Participants were unanimous in describing that disclosures of abuse from older adults most often occurred within contexts not necessarily related to deliberate abuse reporting and were more likely to occur as the trusting relationship became better established. “*It’s not generally like, a lot of outright disclosures; it doesn’t really happen that way. It’s more you’re going through, you’re gathering some history and trying to understand what the struggles are with that person, and you come to learn about the relationship between them and their adult child. You come to learn the certain things have happened where it could have been financial and/or emotional abuse.*” (Urban/rural Seniors’ Agency Coordinator).

The stories of abuse would typically emerge incrementally. “*They might…just mention a little bit, because sometimes they might be thinking ‘Oh yeah, it’s nothing, it’s nothing, right?’... I think it is more of a just a little quiet conversation…This is happening to me. And they probably won’t even give you the full details right away, either. You’d have to be probing for some more*.” (Rural Homecare Social Worker).

Providers also recognized that small steps on the client’s part were a success, given the often fragile and vulnerable state of the older adult. “*The first disclosure sometimes can just have so much emotion with it….they don’t have the ability to take any action or take any steps.”* (Urban Hospital Social Worker) The value of ongoing discussions, directed at the pace desired by the older adults, also highlighted the importance of incremental steps. “*The more that people are open and talking – even if they don’t make any changes – they’ve acknowledged it, they’ve said it out loud and that’s a huge step for them*.”(Urban Community Social Worker).

Incremental improvement in overall well-being could be a pathway to increasing the older adult’s ability to deal in the most effective way possible for them with the abuse they experienced. Once the client’s most pressing health and social needs had been addressed, participants suggested there may be an increased “readiness” to move forward. “*In our program, we help people become physically stronger. We know they’re eating better; we know they’re thinking more clearly because their medications have been adjusted. It’s when all of those things happen that people then can start thinking, ‘Okay, we need to do something about this. I need to do something about this. I know who I can trust. I know what I’m going to do next.*’” (Urban Hospital Social Worker).

### Individualism

Recognizing that each older adult presents with unique needs and strengths, individualism as a harm reduction principle highlights the importance of tailoring messages, and maximizing treatment options [30]. Participants described the range of emotions exhibited by older adults experiencing abuse and the frequent reluctance or inability to find a way out of their situation. “*Bone deep fear…fear is huge. Embarrassment... Blame. Guilt. …That’s why they rarely report it, because they are trying to deal with all of these emotions that are coming up with it - and hoping it will just go away. And trying to figure out other ways that they can make it go away, without dealing with it”*. (Rural Abuse-specific Agency) Participants also reported diverse responses from the older adults that required them to adapt and adjust their therapeutic approaches. The following illustrates a potential range of responses from older adults. “*It’s not okay that your son takes your money from your pension, because you need to buy your medication for your diabetes…And sometimes they shrug their shoulders, sometimes they cry. And then sometimes they shut down and you’ll never hear the conversation again*.” (Urban Community Health Nurse).

Affirming the decisions of the older adult and offering support to try to deal with their situation was offered to maximize treatment options for participants. “*So you might choose to stay. That you don’t need to blame yourself. You don’t need to accept it because you stay. Staying might feel like a better choice than leaving.*” (Urban Community Social Worker).

### Pragmatism

Recognizing that abused older adults’ attitudes, behaviors and choices were influenced by myriad social and cultural influences, providers relied on listening to the expressed values of the individual. Pragmatism, according to Hawk et al.’s model [30], means that abstinence from harmful behaviors (which, in the case of elder abuse, could mean continuing to choose to live their lives at risk for further abuse) is neither prioritized nor assumed to be the goal of the client.

Participants repeatedly remarked the goal of maintaining family ties often superseded apprehensions about their own safety for many older adults experiencing abuse. “[They’re] *worried that they may lose connection to family… that one person is the only one that they have left to meet their needs*.” (Urban General Seniors’ Services) “‘*He’s all I have, so I know he only cares about me if he gets my money and he’s using me but, if I don’t have him, who do I have? Nobody. I can’t take my money with me when I die, so I’ll continue to let him use me, take advantage of me, because I would rather do that than be alone.’ And I think that’s the horrid reality for a lot of people*.” (Urban Hospital Social Worker).

In many circumstances, the goals of the individual older adult reflected their overarching concern for their adult children. When family members were the perpetrators of the abuse, providers recognized how the bonds of love and kinship could push the older adult to not disclose abuse for the welfare of both the family member as well as for themselves. “*When adult children are involved, they tend not to want to necessarily punish…They’re often seeking help for that adult child because they feel that person is very troubled…[their child] wouldn’t be doing this if they didn’t have real problems of their own… addiction, or mental illness, or unemployment, financial issues…Its not ‘I need help’- it’s my adult child, I want help for them*.” (Urban Community Physician).

### Autonomy

The principle of autonomy, in which professionals recognized that clients ultimately make their own choices based on their own abilities, resources, beliefs and priorities [30], was a dominant theme in the data. References to autonomy were most often described in relation to the client decision-making process following disclosure. Providing education, suggestions and support were pivotal resources used by providers to empower older adults experiencing abuse to consider the range of options available to them and inform their autonomous decision. “*What I would normally do is go through a generic idea of here would be some of your options or their options for how you could move forward, and then I would suggest to them the next kind of steps*.” (Urban/rural Seniors’ Agency Coordinator).

Participants were clear that autonomy was foundational in their work with older adults experiencing abuse. There was ongoing reinforcement that providers would support and provide suggestions, but ultimately, the decisions that needed to be made rested with the client. “*It has to be their call as to whether or not they move forward to make the situation better. We have to be very careful as people who might be responding to situations of elder abuse that we don’t replicate the abuse by forcing what we think is best for them on them*.” (Rural Abuse-Specific Agency Coordinator) Not only was the decision about next steps the older adult’s to make, the pace at which the next steps was also directed by the client. “*If they choose not to report it right now, it can still be reported in the future - like when they are ready*”. (Urban/rural Seniors’ Agency Coordinator).

Resolving the tension between promoting autonomy and protecting older adults in situations of potentially impaired capacity was described as an ongoing challenge, particularly when some professionals involved might not have complete information about the clients’ circumstances. Frustration was expressed by a number of participants in relation to this issue: “*This self-determination stuff. Like they’re saying that it’s up to these people to make like their decisions, but they’ve been determined to be not capable to making those specific decisions that you’re saying they’re able to do*.” (Rural Abuse-specific Agency Social Worker) “*There’s so many shades of grey…this person can make the decision for X, but not Y and Z…So they’re making these determinations on what kind of decisions they can make and then, when it comes to the really tough stuff, nobody wants to make that really tough decision on that person. Like we’ve seen that with the public trustee’s office… taking an arm’s length approach*.” (Urban Police Services Social Worker).

Of the six harm reduction principles, recognizing the autonomy of the older adult client to make choices that placed them at risk seemed to create the most moral distress for the providers interviewed. “*You work really, really hard trying to put all of the pieces together for the senior, and then they say, “Nope, I’m going back to my family”. And it’s very difficult sometimes*”. (Rural Abuse-Specific Agency Coordinator).

### Accountability without termination

This harm reduction principle is based on the premise that clients are responsible for their own decisions, but that they are not abandoned by the providers involved with them, regardless of the decisions that the older adult makes. “*Sometimes you work so hard to try to find a solution for them, and then at the end of the day, they end up going backwards again. I think it’s critical…to not just give up on them if they make that decision*.” (Rural Abuse-Specific Agency Coordinator).

This principle was enacted by providers making arrangements for ongoing contact and check-ins. “*Just keeping the door open in case things change…let them know you’re going to come back to visit and let them know that just because they don’t want to report this abuse that you know you haven’t forgot about them necessarily”* (Urban Police Services Social Worker).

## Discussion

While the potential value of incorporating harm reduction principles into services for older adults experiencing abuse is still emerging in the literature [5], this paper has demonstrated that service providers do make use of these principles on a regular basis. Our analysis illustrated a clear congruence between each of Hawk and colleagues’ [30] six harm reduction principles and the approaches reflected in the narratives of professionals who provided services to this population. No additional principles were identified from the thematic analysis.

Each of the harm reduction principles contributed in meaningful ways to providers’ practice in working with these vulnerable older adults, although some might be emphasized differentially at different phases of the disclosure and intervention process. Enactment of a humanistic approach formed the basis of the therapeutic client-provider relationships and could be considered the basis from which all else proceeded. Quinn and Tomita [34] noted that the powerlessness and learned helplessness inherent in situations of abuse reinforce the perception that older adults can do little to change their circumstance, and thus they may give up seeking or accepting help. Economic, interpersonal, and functional dependencies of abused older adults have been associated with feelings of powerlessness, helplessness and a lack of capacity to challenge the abuser [35]. Harm reduction approaches can help to challenge these beliefs.

References to supporting autonomy figured prominently in the narratives. Autonomy was well-recognized as central to working with older adults experiencing abuse. Within the context of a trusted relationship, abuse is recognized to disrupt autonomy, potentially leading to limitations in agency regarding personal welfare, finances, thoughts and emotion [36]. In the case of adult children perpetrators of abuse, older adults have been demonstrated to often choose to maintain the family relationship in spite of personal risk [37, 38]

While participants were in agreement that respect for the older adults’ autonomy was paramount, there was evidence that some providers felt distress when older adults did not engage with services or chose to return to a high-risk environment. Such choices are relatively common, with 13–58% of abused older adults refusing service completely, and only 16–28% pursuing all of the recommended interventions [39, 40]. One study on healthcare workers’ attitudes towards clients receiving harm reduction services for addictions suggested that the morale of staff could be negatively affected by the relentless exposure to suffering [41]. Service provider distress appeared elevated when there was uncertainty about the capacity of the older adult to make a truly informed decisions. Given that only about half of persons who meet the criteria for dementia have received this diagnosis from a clinician [42], there is legitimate concern about the default to autonomy in some cases of older adult abuse. Improved access to timely assessments of capacity in cases of abuse of older adults would facilitate the development of tailored intervention plans. Better integration of abuse, clinical, and legal services has long been advocated, but seems far from being realized.

Explicit incorporation of the principles of harm reduction into services for abused older adults has the potential to provide additional clarity and direction to service providers, resulting in a less disjointed approach. Both undergraduate and continuing professional education on harm reduction for service providers working with abused older adults is needed. Opportunities to discuss common ethical dilemmas and promising practices related to providing services to abused older adults may mitigate the emotional toll experienced by service providers. In addition, developing strategies to educate older adults about how and why harm reduction approaches can be useful in situation of abuse can better empower clients in making their decisions.

### Strengths and limitations

The sample of providers included social work, nursing, medicine, justice, and program coordinators from a broad range of services across three Prairie provinces in Canada, including urban, rural and urban-rural mixed settings. The analysis process demonstrated rigor, provided extensive documentation of data, and the potential for transferability to other settings in which older adults experience abuse.

The principles of harm reduction were operationally defined by Hawk and colleagues [30], but because, to our knowledge, this is the first time where they have been specifically applied to older adult abuse, there remains substantive work to be done in refining and expanding the definitions of the principles specifically to abuse of this population. Although explicit questions about harm reduction approaches were not included in the interview, analysis of the data revealed themes consistent with harm reduction approaches. Future studies might specifically investigate providers’ use of harm reduction principles along the entire journey of working with diverse older adults experiencing abuse and examine the outcomes of promoting autonomy in cases where capacity is uncertain. In addition, exploring the perspectives of older adults experiencing abuse on harm reduction approaches used by providers would make a substantive contribution to the evidence in this area.

## Conclusions

The principles of harm reduction described by Hawk et al. [30] provide a useful lens for examining providers’ work with older adults experiencing abuse. While further refinement of the operational definitions of harm reduction principles specific to their application with older adults is still required, this framework aligns well with both the ethical imperatives and the practical realities of supporting older adults experiencing abuse. Particularly in light of evidence that abuse of older adults appears to be increasing due to external events such as COVID-19, efforts to optimize practices that support older adults experiencing abuse are timely [43]. This paper contributes to the evolving developments in this area by providing specific examples of harm reduction approaches can be helpful in working with abused older adults.

## Supplementary Information

**Additional file 1.**

## Data Availability

The datasets generated and/or analyzed as part of the current study are not publicly available due to privacy and ethical obligations, but may be available from the corresponding author on reasonable request.
